# KLF feedback loops in innate immunity

**DOI:** 10.3389/fimmu.2025.1606277

**Published:** 2025-06-04

**Authors:** Jessica M. Salmon, Holly Adams, Graham W. Magor, Andrew C. Perkins

**Affiliations:** Australian Centre for Blood Diseases, School of Translational Medicine, Monash University, Melbourne, VIC, Australia

**Keywords:** macrophage, inflammation, KLF4, KLF3, transcriptional regulation, feedback loops, chronic inflammatory disease, innate immunity

## Abstract

The Krüppel-like factor (KLF) family of zinc finger transcription factors regulate the expression of genes involved in a wide range of cellular processes, including cell proliferation and differentiation. In haematopoiesis, KLFs have essential roles in myeloid cell differentiation and function. KLF4 is a critical regulator of macrophage development and initiates pro- and anti-inflammatory signalling pathways in response to various stimuli. KLF2, KLF3 and KLF6 also play important roles in regulating these pathways. Here we review how KLFs cooperate and compete to either activate or repress target genes to influence initiation and resolution of inflammatory responses in macrophages. We also discuss how KLFs may be involved in the development of chronic inflammatory conditions.

## 1 Introduction

Innate immune cells, particularly within the spleen, skin, gut, and lungs, are a key component of the immune system. They are the first line of defence against invading pathogens, or first responders to tissue injury (1). Macrophages, dendritic cells (DC), mast cells, neutrophils and natural killer (NK) cells are all considered to be part of the innate immune system, reacting rapidly to inflammatory stimuli, and driving adaptive processes that result in long-term immunity. Detection of a pathogen, or other immune signal, occurs through receptors on both the cell surface and within the cytoplasm; these initiate a cascade of signalling and transcriptional changes that result in the secretion of pro-inflammatory molecules; including potent cytokines and chemokines (2). It is these molecules, particularly produced by monocytes or tissue-resident macrophages, that engage other cells of the innate and adaptive immune system to synergistically mount an immune response. Ultimately, this results in the clearance of the pathogen, resolution of the inflammatory stimulus, tissue repair and a return to homeostasis (3).

There is still much to learn about the transcription factor (TF) networks that initiate pro-inflammatory signals and those that keep the immune system in check. Herein we review what is known about the transcriptional feedback loops that exist between different members of the Krüppel-like factor (KLF) TF family in macrophages during differentiation, homeostasis, inflammation, macrophage polarisation and trained immunity. We focus on KLF4 and its possible interactions between KLF2, KLF3 and KLF6.

## 2 The Krüppel-like transcription factors

### 2.1 KLF structure

**Figure 1:**
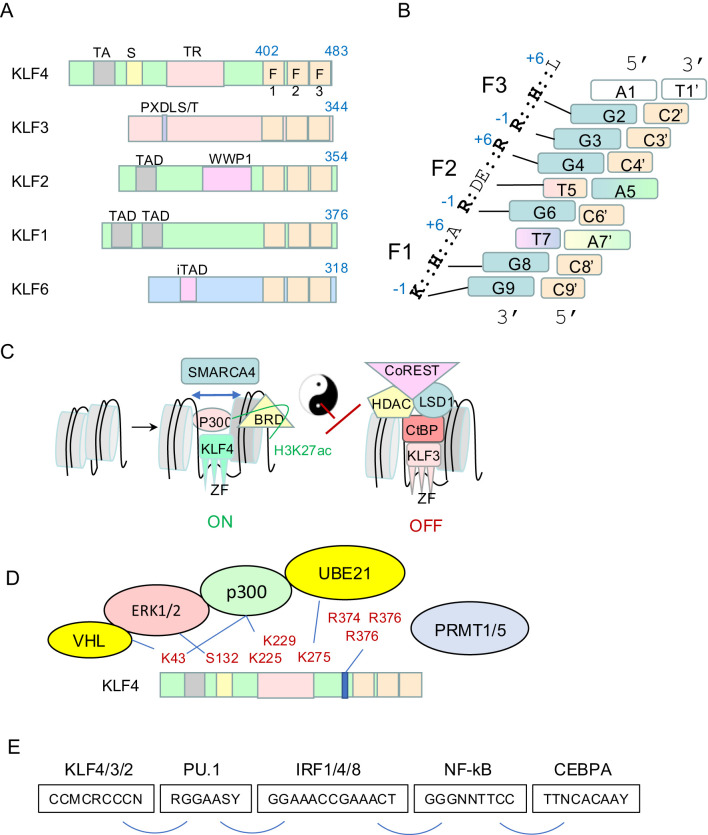
Biochemistry of the KLF family. **(A)** Schematic of murine KLF4, KLF3, KLF2, KLF1 and KLF6. The DNA-binding domain of three C2H2 zinc fingers is at the very C-terminus. The N-terminus contains well defined domains and motifs that recruit different cofactors such as p300/CBP, CtBPs, and others. **(B)** The DNA binding domain of all SP/KLFs binds an 8-9bp motif *in vitro* and *in vivo*. This model is a composite derived from the structures of SP1 and KLF4 bound to different dsDNA molecules (5, 6). Each nucleotide is color-coded. Redundancy of binding specificity at positions 7 and 5 on the G-rich strand is indicated by colour grading. Position 9 on the G-rich strand is white to reflect redundancy for any nucleotide for the KLF4 clade but specificity for a G for SP factors and some KLFs. The key amino acids in ZF1, ZF2 and ZF3 (relative to an alpha helix) of murine KLF4 and the nucleotides they contact are indicated. **(C)** Group 2 KLFs (KLF1/2/4/5/6/7) recruit p300/CBP and associated proteins to acetylate chromatin tails (H3K27ac) and well as lysines in KLF4. They recruit SMARCA4 and associated SWI/SNF proteins to open chromatin and pioneer for other transcription factors. Group 1 KLFs (KLF3/8/12) recruit CtBP1/2 and subsequently HDACs to deacetylate chromatin, as well as complexes containing LSD1 and Co-REST. **(D)** KLF4 post-translational modifications are critical for responses to external signals and effector functions. ERK1/2 phosphorylate KLF4, E3 ubiquitin ligases induce lysine ubiquitination and subsequent degradation and PRMT1/5 induce arginine dim-methylation which has functional consequences. **(E)** KLFs bind cis regulator modules in enhancers and promoters with a specific syntax with respect to other transcription factors such as PU.1, CEBPA, IRFs, and REL/NF-κB to co-ordinate gene expression in macrophages and other cell types. These motifs are found in different combinations within KLF4-regulated enhancers and promoters of macrophage genes.

The Krüppel-like factor (KLF) family of 17 TFs are widely expressed (4) and functionally critical in most mammalian cell types. The KLF family shares structural homology and DNA-binding similarities to the 8 members of the specificity protein (SP) transcription factors, which are often collectively referred to as the SP/KLF family; there are 25 members in total. SP/KLF TFs are defined by three DNA-binding C_2_H_2_ zinc fingers located at the C-terminus; the zinc fingers (ZFs) are linked by two conserved TGEKP sequences (**Figure 1A**). Each ZF interacts with three consecutive nucleotides on the G-rich strand in the major groove of DNA (7), although much of the literature refers to binding site specificity on the C-rich strand (**Figure 1B**). Thus, the DNA-binding specificity *in vitro* and *in vivo* is similar for all SP/KLF factors.

Many *ex vivo* DNA-binding experiments, as well as *in vivo* chromatin immunoprecipitation sequencing (ChIP-seq) experiments, have shown KLFs bind best to a CCM-CRC-CCN DNA motif (on the C-rich strand), where M=C or A, and R=A or C (8–11) (**Figure 1B**). This motif fits very well with the NMR structure of SP1 bound to DNA (5), and the crystal structure of KLF4 bound to DNA (6). For each zinc finger, a conserved lysine or arginine at the -1 position, relative to an alpha helix, makes hydrogen bonds with guanines on the G-rich strand at positions G9, G6 and G3 for F1, F2 and F3, respectively (6) (**Figure 1B**). Conserved amino acids at the +3 positions in F1, F2 and F3 make hydrogen bonds with G8, T5 and likely G2 based on the structures of SP1 (5). This latter interaction was not observed in the crystal structure of KLF4, but this is likely due to the fact an atypical dsDNA sequence was chosen for crystallisation (with an A at this position 2 on the G-rich strand). It is unlikely KLF4 binds such a sequence *in vivo* based upon motif analyses of ChIP-seq datasets (9). There is no direct binding between the alanine at +6 in F1 and DNA, so this explains the redundancy at this site with respect to *in vivo* binding specificity (12) (**Figure 1B**). The conserved +6 arginine in F2 makes hydrogen bonds with G4 in all SP/KLF family members, but the +6 amino acid in F3 differs between SP family members and some of the KLFs. It is a lysine in SP1 to SP8; and it makes an important hydrogen bond with G1 in the 9bp SP1-bound motif (5). This amino acid is a leucine in KLF1–8 and KLF12, 16 and 17, so it cannot form such a bond, but it is a lysine in KLF9–11 and KLF13-15. Thus, there is no specificity for the binding motif at this position for KLF4 type family members, but specificity for a G on the G-rich strand for all SP family members and half of the KLFs. Hence, we have not coloured this nucleotide in our KLF4 binding model (**Figure 1B**).

In summary, SP and some KLF members bind a 9bp consensus CCM-CRC-CCC, whereas KLF4 family members bind CCM-CRC-CCN, which is, in effect, just an 8bp consensus. This suggests KLF4/3/6 subfamilies should bind in principle to four times as many sites in the genome, but all at slightly lower affinities that than SP family. This might have important implications for competitive binding interactions that is worth further investigations. Lastly, binding of F2 to the central GTG sequence is quite interesting. There are direct interactions between the conserved glutamate at +3 in F2 and the thymine or cytosine in the solved structures for KLF4 or SP1, respectively (5, 6). Liu et al. have shown affinity of KLF4 for methylated cytosine at this C5 position. In this case the methyl group of ^m^C makes hydrogen bonds in the same way as the methyl group of thymine; *i.e.* it behaves like thymine in the structure. This has important *in vivo* binding implications. It suggests the C at this position that is commonly found by *de novo* motif discovery of KLF ChIP-seq datasets might actually be ^m^C or even ^hm^C in many cases, including as part of a G^m^CG trinucleotide. This could be used to an advantage by KLF4 and related KLFs with respect to being able to bind methylated enhancers and promoters. Alterations in binding affinity at this site are clinically relevant as a rare mutation in human KLF1 at this position (*i.e.* p.E325K) results in altered DNA-binding specificity (13) and autosomal dominant inherited haemolytic anaemia (14).

### 2.2 KLF cofactors and epigenetic gene regulation

The 17 KLFs can be divided into 3 groups based primarily on similarities in the amino-terminal regions. They act as repressors, activators, or both repressors and activators, depending upon different co-factors recruited to DNA via distinct domains (15–17). This results in different biochemical activities, and ultimate action as activators or repressors, depending upon which set of co-factors is engaged. The KLF4 clade (KLF1/2/4) all have well defined N-terminal transactivation domains (TADs) that recruit P300 and/or CBP (**Figure 1C**). P300/CBP is a potent acetyl transferase. It induces the H3K27ac mark on chromatin which is strongly associated with active gene expression. P300/CBP can also acetylate non-histone proteins including KLF4 itself to alter function (see section 2.3). The KLF4 clade of KLFs can also recruit SWI/SNF chromatin remodelling complex components, such as SMARCA4/Brg1 (18). This results in ATP-dependent repositioning of nucleosomes; an activity that is fundamental to pioneering activity and for recruitment of settler co-factors. This process is likely critical for assembly of fully functional enhancers and for co-operative gene regulation (**Figures 1C–E**).

On the other hand, the KLF3/8/12 subclade of KLFs all harbour a conserved PXDLS/T motif (19) (**Figure 1A**), which can recruit the CtBP family of co-repressors, CtBP1 and CtBP2 (**Figure 1C**). These in turn recruit histone deacetylases (HDACs), which de-acetylate H3K27 in chromatin to silence gene expression. CtBPs also recruit a suite of other enzymes such as LSD1, CoREST and NURD complexes that together induce epigenetic changes in histone tails that silence gene expression (**Figure 1C**). Thus, the KLF4 and KLF3 clades have broadly opposing biochemical activities via recruitment of different co-factors. Since KLF3 can compete for binding *in vivo* with the KLF4 clade (20), these biochemical differences result in fine tuning transcriptional outputs. Less is known about the cofactors recruited by the KLF5/6/7 clade of KLFs. They have been reported to behave as transcriptional activators or repressors in different contexts. More work is required to fully understand the biochemical mechanisms by which they work. Lastly, the KLF9-11, 13–15 family can recruit Sin3A to repress transcription (17). These are not very well studied in macrophages so they will not be discussed in detail in this review.

### 2.3 KLF post translational modifications

KLF4 and other members of the KLF family undergo extensive post-translational modifications (PTMs) that are important for regulation of function. Most of this work has been performed in non-macrophage cell types and needs be explored more in macrophages, but there are important key insights from other cell types that are likely applicable. KLF4 is phosphorylated at Ser132 by ERK1/2 (*e.g.* via LIF signalling) in embryonic stem cells (21) (**Figure 1D**). This results in nuclear export and exclusion from a transcriptional interaction with OCT4 and NANOG that maintain the pluripotent state. Thus, ERK or MEK inhibitors can increase nuclear retention and activity of KLF4. Signalling through toll-like receptor 4 (TLR4) in macrophages in response to LPS also results in ERK1/2 signalling, phosphorylation and down regulation of KLF4 (22). These authors showed inhibition of this phosphorylation by MEK inhibitors improved outcomes in a mouse model of sepsis. KLF4/2/1 all recruit P300/CBP which acetylates histone H3 tail at K27 (23). This provides a permissive environment for active transcription. Recruited P300 can also acetylate K43 in KLF4, and thereby inhibit ERK signalling and increase KLF4 stability (23).

KLF4 is sumolyated by Ubc9 (UBE21) at a site in the ‘repression domain’ that conforms to the classical consensus sequence ψK*X*E, where ψ is a bulky hydrophobic amino acid (such as Ile, Leu, and Val), and *X* is any residue. This leads to engagement with the ubiquitin ligase pathway and KLF4 degradation. Thus, loss of this site in KLF4 increases its stability and potency as a reprograming factor (24). KLF4 is also sumolyated in macrophages in response to IL-4 signalling and this results in polarisation from an M1 to M2 state (25) (see section 3). The VHL E3 ubiquitin ligase can also recognise K43 in KLF4 and lead to its degradation in other contexts (26) (**Figure 1D**).

KLF4 is methylated on arginines that lie just upstream of the zinc finger domain by the arginine di-methyl transferases, PRMT1 and PRMT5, in different systems (**Figure 1D**). Methylation of Arg-396 by Prmt1 in ES cells is important for repressing primitive endoderm differentiation in favour of pluripotency (27). Methylation by PRMT5 at these same three arginines results in a conformational change and reduced ubiquitination and degradation by the VHL ubiquitin ligase pathway in macrophages, thus increasing the stability of KLF4 (28).

The cofactors that are recruited by CtBPs include histone modifying enzymes that introduce repressive histone modifications, and co-factors which influence the post-translational modifications of CtBPs by sumolyation. Examples of such co-factors include histone methyltransferases (EHMT 1/2), lysine-specific demethylase (LSD1), histone deacetylases (HDAC 1/2) (29, 30), members of the polycomb repressor complex, SUMO E2 conjugating enzymes and E3 ligases (31). CtBPs may also harbour their own enzymatic activity to influence chromatin structure (19). The combined effect of these multiple binding partners is the remodelling of chromatin to alter DNA accessibility and silence gene expression.

In summary, KLF4, KLF3 (and probably all KLFs) undergo extensive PTMs that alter protein stability, nuclear localisation and function. Many of these pathways are under explored during macrophage responses to inflammatory signals, and many are targetable by small molecule inhibitors (see Discussion).

### 2.4 KLF transcription factor partnerships at myeloid gene enhancers and promoters

Inflammatory gene expression signatures differ depending on context. The TFs that are induced following an inflammatory stimulus (stimulus-induced) belong to several main families (32, 33): the interferon regulatory factors (IRFs), signal transducers and activators of transcription (STATs), the nuclear factor of the κ light chain enhancer of B-cells (NF-κBs), CCAAT enhancer binding proteins (CEBP), and members of the activator protein (AP1) complex, FOS and JUN (32, 33) (**Figure 1E**). The coordinated expression and interaction of these inflammation-responsive TFs with cell type specific (lineage-determining) TFs ultimately determines what genes will be expressed (33). The myeloid lineage-defining TF, PU.1, acts as a pioneer factor to mark myeloid specific enhancers (34). At the gene level, these PU.1-bound enhancers become “poised” for the recruitment of inflammatory TFs and the rapid induction of expression of target genes (34). Mapping of regulatory elements for monocytes and tissue resident macrophages identified the PU.1 binding motif in all active regions, but found CACCC-box elements to be more highly enriched in the active enhancers of circulating monocytes (35). This also correlated with high levels of *Klf4* expression and supports previous observations that KLF4 is important for monocyte differentiation (36). Multiple TF binding sites exist within these regions, and not all genes bound by PU.1 are activated by the same stimulus (37). It is therefore evident that interactions between multiple transcription factors lead to the activation or repression of specific gene sets and that KLF4 and other KLFs may be involved in the activation and polarisation of circulating monocytes and migration to tissues (**Figure 1E**).

### 2.5 KLF transcriptional networks

**Figure 2:**
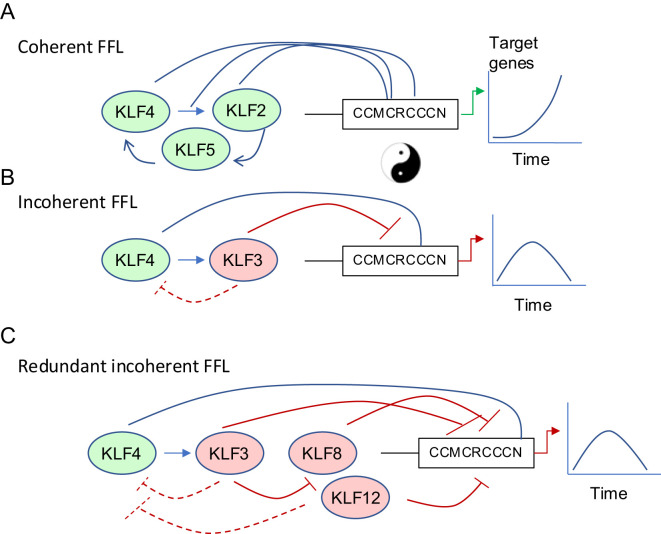
Coherent and Incoherent Feed Forwards Loops regulate transcription of target genes in a dynamic tuneable fashion. **(A)** KLFs can work in coherent feed-forward networks to amplify gene expression (e.g. KLF2/4/5 in ES cell pluripotency maintenance) (44) **(B)** KLFs can work in incoherent feed-forward loops to initiate a transient gene expression program followed by silencing after a period of time (20, 45). **(C)** There is redundancy in negative regulation of KLF networks. KLF3 can repress KLF8 and KLF12, which are themselves negative regulators of the upstream positive and negative regulators of target gene expression (45).

The KLF family plays important roles at almost every stage of mammalian cell development. From the inner cell mass stage onwards, many SP/KLF factors are expressed in the same cells (4, 9, 38). Mouse genetic studies have demonstrated critical roles for KLFs 5, 2, 6 and 4 at different stages of embryo, or early post-natal development (39–43). Studies *in vitro*, using embryonic stem (ES) cells, reveal that KLF2, 4 and 5 have very similar DNA-binding profiles and that there is functional redundancy between these factors. All three bind to enhancers and promoters in each other’s genes (by ChIP-seq) to work in a coherent feed forward loop (CFFL) that maintains ES cell pluripotency (**Figure 2A**). There is therefore significant redundancy with respect to maintenance of ES cell potency; only triple knockdown of KLF2/4 and 5 led to loss of pluripotency and differentiation of ES cells in the presence of ongoing LIF stimulation (44). The *Klf1* knockout mouse has a striking phenotype of embryonic lethality at embryonic day (E)14.5 due to severe anaemia (46). Interestingly, *Klf3* and *Klf8* are both direct and indirect (via KLF3) targets of KLF1 (47), and KLF3 represses both *Klf1* and *Klf8* in a negative feedback loop (45, 48). In *Klf3* knockout mice, *Klf8* is upregulated in multiple tissues (49), but particularly in erythroid cells. Deletion of *Klf8* results in minimal expression changes, perhaps due to functional redundancy between KLF3 and KLF8 and/or KLF12. Indeed, deletion of both *Klf3* and *Klf8* in mice results in further de-repression of genes compared to *Klf3* knockout alone, and embryonic lethality (49). *Klf8* and *Klf12* are both markedly induced when *Klf3* is deleted in various cell types (45). These two poorly studied KLFs belong to the same clade as KLF3 and act predominantly as repressors (50). This type of transcriptional network (*i.e.* repression of a repressor that acts on a common downstream target) ensures there is a repressive KLF available to the cell even when one or two are damaged or deleted; *i.e.* there is extensive network redundancy that becomes obvious in double knockout mice or combined heterozygous and homozygous knockout mice for *Klf3* and *Klf8* (49) (**Figures 2B, C**). Together these data demonstrate how KLFs can oppose the function of, or assist/enhance one another, and that redundancy also exists between KLFs with similar function. It is very likely similar networks are at play and not yet well studied in macrophages.

## 3 KLFs in macrophage differentiation

### 3.1 Lineage specification

**Figure 3:**
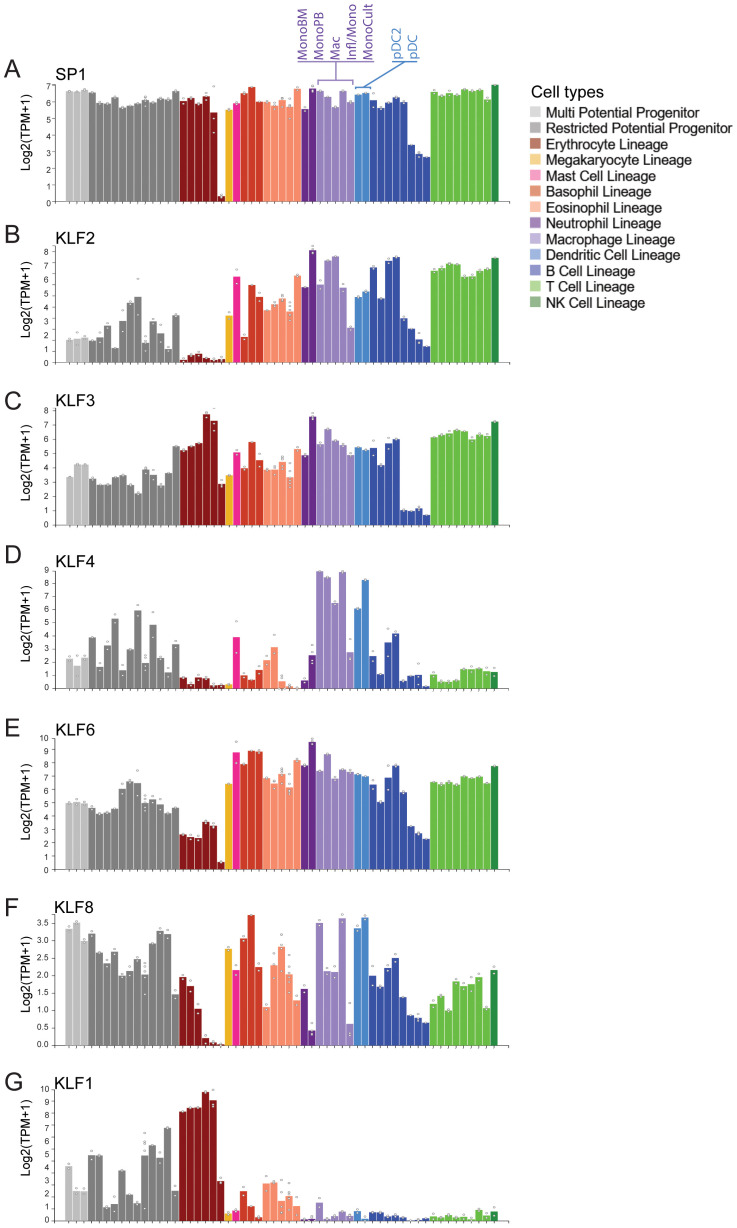
Expression of many KLF/SP family members in monocytes and macrophages. **(A)** Data mining from the Hemosphere online RNA-seq database derived from (51). SP1 is ubiquitously expressed in all blood cell lineages. **(B–F)** KLFs 2, 3, 4 and 6 are all highly expressed in FACS-sorted monocytes and macrophages from the blood and bone marrow. On the other hand, KLF8 is expressed at low levels in all blood types, KLF3 is also high in erythroid cells whereas KLF4, KLF2 and KLF6 is not. **(G)** KLF1 is highly expressed in erythroid cell but not in monocytes.

**Figure 4:**
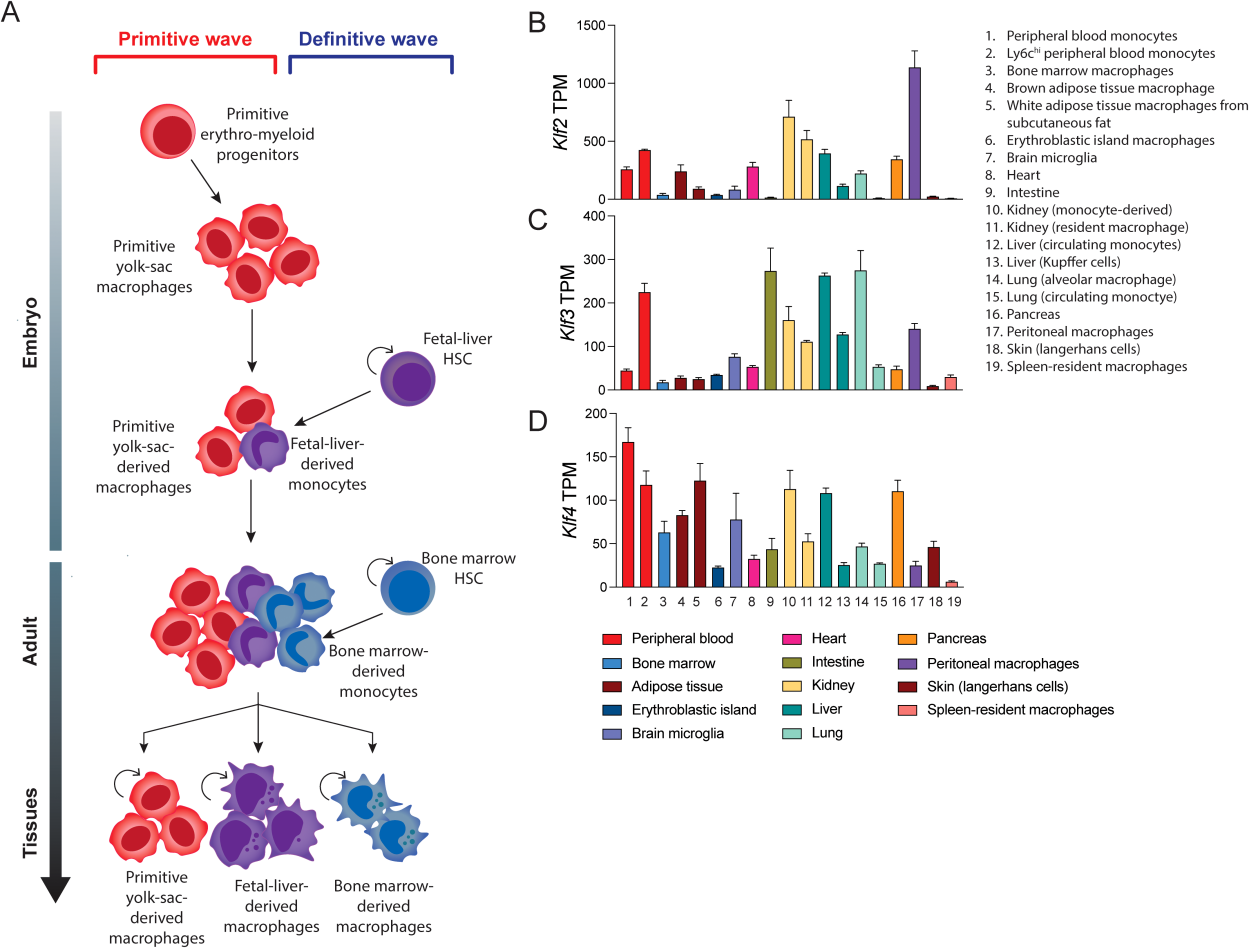
Monocyte/macrophage differentiation and KLF expression in monocytes and tissue-resident macrophages. **(A)** The stages of macrophage development. Yolk-sac erythro-myeloid progenitors differentiate into primitive yolk-sac derived macrophages that are long-lived. Foetal liver and bone marrow derived hematopoietic stem cells (HSCs) give rise to short-lived and long-lived macrophages during development and adulthood. A combination of these different macrophage sources contribute to the populations of adult tissue-resident macrophages **(B–D)** Data mining for a suite of different GEO submissions of different macrophage datasets for KLF expression. This bioinformatic analysis of these diverse datasets was undertaken by (53). It is difficult to compare absolute expression levels of different KLFs but relative expression of KLF2, KLF4 and KLF2 shows marked differences. For example, KLF3 is relatively highly expressed in alveolar macrophages and gut-derived macrophages.

Our analysis of publicly available RNA-seq data sets from murine blood cell types shows some SP/KLF family members such as SP1 are very highly expressed in all cell types (**Figure 3A**). On the other hand, KLF4, KLF2, KLF3 and KLF6 are all highly expressed in monocytes, macrophages and dendritic cells, whereas KLF1 is restricted to erythroid cells (**Figures 3B–G**) (51). Macrophages are generated from haematopoietic stem (HSC) and progenitor cells during two waves of ontogeny (52). The earliest detection of macrophages occurs during embryonic development from primitive macrophage progenitors in the yolk sac and later from definitive HSCs and multipotent progenitors in the foetal liver and adult bone marrow (**Figure 4A**). Studies in mice have demonstrated that most tissue-resident macrophages migrate to the organs early during development (54, 55). The differentiation, proliferation, and survival of circulating monocytes or tissue-resident macrophages is driven by both the expression of lineage defining transcription factors, such as PU.1, and the instructive signals of cytokines, notably macrophage colony stimulating factor (M-CSF/CSF1). Monocytes in the circulation mature and migrate to the tissue to become tissue-resident macrophages. These are often the first line of defence against an invading pathogen, and are also critical to the maintenance of homeostasis and the removal of debris or damaged tissue.

A recent study which included extensive mining of expression data from macrophages isolated from different tissues shows expression of KLF4, KLF2 and KLF3 in different ratios (**Figures 4B–D**) (53, 56–69). Another recent paper suggests expression of KLFs in tissue-resident macrophages can change significantly depending on the local inflammatory and signals. Furthermore, different KLFs are essential for macrophage persistence in different sites/niches (70). Thus, under certain conditions essential requirements for specific KLFs becomes apparent in macrophages.

### 3.2 Differentiation of macrophages from the granulocyte-macrophage progenitor

**Figure 5:**
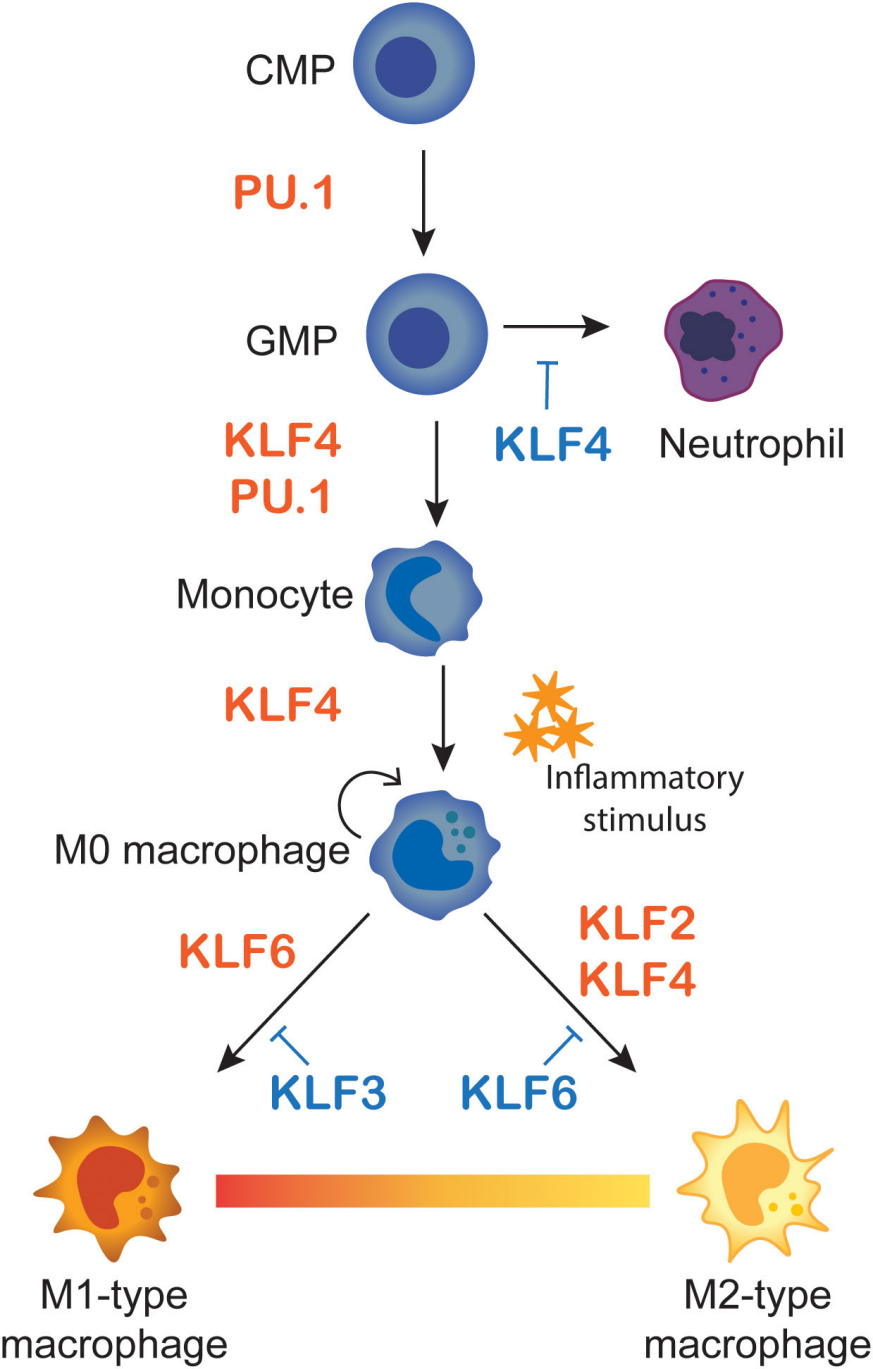
Macrophage differentiation and M1/M2 polarisation. A Summary of KLF influences on monocyte differentiation and macrophage polarisation. Monocytes differentiate from common myeloid progenitors (CMP) and granulocyte-macrophage progenitors (GMP), where KLF4 favours monocyte over neutrophil lineages. Monocytes then migrate to tissues as naïve M0-type macrophages. Upon inflammatory stimulus they polarise to M1-type pro-inflammatory or M2-anti-inflammatory macrophages. The influences of KLFs on the different stages of differentiation and polarisation are summarised.

KLF4 is a critical regulator for monocyte differentiation from common myeloid progenitors (CMP) (**Figure 5**). *Klf4* knockout mice die shortly after birth due to defective skin barrier formation (39), but these mice have relatively normal hematopoietic development. However, conditional deletion of *Klf4* in adult CMPs and differentiation *in vitro* results in a strong granulocyte bias at the expense of macrophages, with no apparent defects in megakaryocyte or erythroid differentiation (36). This suggests that KLF4 is involved in the differentiation from the shared granulocyte-macrophage progenitor (GMP). In contrast, CMPs overexpressing *Klf4* have a significant increase in macrophage output, and subsequent decrease in granulocyte differentiation, using the same culture conditions (36). Similarly, differentiation of HL-60 human pro-myelocytic cells overexpressing *KLF4* results in a marked increase in monocyte differentiation and a reduction in granulocyte differentiation (36, 71). Interestingly, the monocyte differentiation block seen in PU.1 null hematopoietic cells can be rescued by ectopic KLF4 expression. *Klf4* expression is absent in PU.1 null cells, and together with elegant biochemical studies, Feinberg et al. demonstrated that KLF4 is a critical downstream effector of PU.1, specifically for the macrophage lineage (**Figures 1E** and **5**) (36). Furthermore, recipient mice transplanted with *Klf4* knockout foetal liver cells were found to have defects in macrophage differentiation and lacked splenic inflammatory macrophages (71), suggesting that KLF4 is not only important for the initial specification of the monocyte lineage, but also for the downstream function of tissue-resident macrophages. IRF8 is another transcription factor that cooperates PU.1 and KLF4 during GMP differentiation and macrophage maturation. *Irf8* knockout cells also lack *Klf4* expression and IRF8 and PU.1 likely upregulate *Klf4*, and indeed also *Klf6*, via ETS-IRF tandem motifs (72). (**Figure 1E**). These interactions continue to be important during macrophage activation and the expression of pro-inflammatory genes, which will be discussed later.

While KLF4 is important for specification of monocytes, other KLFs do not seem to be important at this stage. Conditional deletion of *Klf2* in mice does not perturb the numbers of neutrophils or monocytes (70). Likewise, conditional deletion of *Klf6* in myeloid cells results in no differences in granulocyte numbers, but a slight increase in monocytes (73). *Klf3* knockout mice have increased numbers of all white blood cell types and inflammatory macrophages (74). While these KLFs are not essential for the differentiation of the monocyte lineage, later studies have demonstrated their importance for regulating the inflammatory response and in the specification of pro-and anti-inflammatory macrophages.

## 4 A network of KLFs influence immune responses

### 4.1 Inflammatory activation

Macrophages and other innate immune cells express pattern recognition receptors (PRRs) that detect molecules produced and/or secreted by invading pathogens or damaged tissue. These include Toll-like receptors (TLRs), retinoic acid inducible gene I (RIG-I)-like receptors (RLRs) and nucleotide-binding domain and leucine-rich repeat containing molecules (NLRs). PRRs detect pathogen-associated molecular pattern molecules (PAMPs), which are produced by pathogens, or damaged cells (damage-associated molecular patterns, DAMPS) (75). One of the most well studied PAMP is LPS, which is produced by gram negative bacteria and is recognised by Toll-like receptor 4 (TLR4) (75). Signalling through PRRs results in the initiation of a rapid inflammatory response. This includes the proliferation and mobilisation of inflammatory macrophages, the production of cytokines and chemokines to recruit additional inflammatory cells, and the clearance of the invading pathogen and repair to damaged tissue (2, 3).

There have been detailed studies of the transcriptional responses to LPS in macrophages, particularly in primary CD14+ macrophages. These studies, using Cap-analysis gene expression (CAGE)-based transcription profiling, show distinct clusters of gene induction and silencing over 48 hours (76). Immediate early transcription factors are induced within the first 30 minutes of LPS exposure. These include *FOS*, *JUN*, *EGR1*-*3*, and *NFKBIZ*, which encodes IκB-δ, a factor involved in NF-κB activation. There is a suite of inflammatory cytokines that is induced a little later (120–180 minutes) and then interferon response genes later again (76). This analysis also revealed the dynamic expression of KLFs after an inflammatory stimulus. *KLF2* is rapidly induced in response to LPS, then suppressed in a manner similar to other immediate early genes with a peak at 45–60 minutes after LPS stimulation (76). *KLF4* induction is slightly delayed (150–180 minutes), and *KLF3* peaks later at 6–8 hours. These results are consistent with our understanding of KLF feed-forward and feed-back circuits (**Figure 2**), which have been described in other cell types (20, 44, 49). In short, early KLF activators are induced, followed by KLF repressors which dampen the response and repress and fine-tune the expression of early response genes.

### 4.2 Macrophage polarisation

Tissue macrophages are long lived and can replicate locally via self-renewal or are replenished by circulating monocytes (52). Naïve, unstimulated macrophages (M0-type) can differentiate into either pro- or anti-inflammatory type cells, depending on the stimulus (**Figure 5**): M1-type macrophages are pro-inflammatory and produce inflammatory cytokines (*e.g.* TNFα and IL-1β); M2-type macrophages are anti-inflammatory, highly phagocytic, and produce non-inflammatory cytokines (*e.g.* IL-4 and IL-10). Many researchers consider these classifications to be overly simplistic. It is more likely that macrophage polarisation is not a fixed state, and that many cells may exhibit both M1- and M2-type characteristics (77). Nevertheless, macrophages can be more or less pro- (M1) or anti- (M2) inflammatory. M1- and M2-type macrophages also display differences in their metabolic processes. M1-type macrophages have increased glycolytic activity. Increased glucose uptake is driven by expression of Hypoxia Inducible Factor 1α (HIF-1α) (78), a component of the HIF-1 TF. Thus, high expression of HIF-1α is closely linked to M1 polarisation. Conversely, M2-type macrophages rely on fatty acid oxidation and OXPHOS, which is supported by the expression of STAT6 and PPARγ (79). Understanding the transcriptional pathways that drive macrophage polarisation is critical to understanding the processes involved in inflammatory disease, wound healing, anti-tumour immunity, and other processes whereby manipulation of macrophage polarisation could have therapeutic implications.

Inflammatory activation is largely driven by activation by the stimulus-induced TF, NF-κB, which cooperates with other TFs (*e.g.* IRF4/IRF8 and STAT1/STAT2) to activate the expression of pro-inflammatory cytokines and chemokines as well as inducible nitric oxide synthase (iNOS), and HIF-1α (63, 80). Other pathways are also activated, including both type I and type II interferon programs (81, 82). Together these pathways facilitate processes involved in cell proliferation, anti-microbial defence and antigen presentation. M2-type macrophages on the other hand are activated via IL-4 and IL-13. This results in STAT6 phosphorylation and dimerisation which results in expression of genes such as *Arg1* and TFs such as PPARγ, which block pro-inflammatory TF activity, and metabolically switch the macrophage to fatty acid oxidation (83) which promotes wound healing and tissue repair.

The role of KLF4 in M1/M2 macrophage polarisation was first documented in 2005; *Klf4* expression is increased in M1-type macrophages (84). KLF4 was found to compete with SMADs (induced by TGFβ signalling) for p300, for which it had a greater affinity (84). In contrast, later studies found KLF4 promoted M2 polarisation whereupon KLF4 binds with STAT6 to produce a M2-type response after stimulation with IL-4 (85). Macrophages deficient in KLF4 had increased M1-type surface markers, antibacterial properties and decreased wound healing abilities (85). Furthermore, SUMOylation was shown to increase KLF4s ability to bind to M2-associated genes such as *Arg1* (63). KLF4 also has been found to bind to several sites in the *Apoe* promoter sequence, causing the upregulation of *Apoe* which can help to switch from a M1-type phenotype to a M2-type phenotype (63). These conflicting reports uncover the complex networks of positive and negative feedback loops that are initiated by KLF4, and further emphasise the importance of examining these networks in the context of other TFs.

Like KLF4, KLF2 is thought to promote anti-inflammatory properties in macrophages. M1-type macrophages have decreased levels of KLF2 and macrophages overexpressing *Klf2* have reduced expression of pro-inflammatory cytokines and dampened anti-bacterial responses (86, 87). Conditional deletion of *Klf2* specifically in macrophages conferred increased protection from microbial infection *in vivo*, primarily through increased iNOS and NO_2_ production, as well increased glycolysis (87). Additionally, mice with *Klf2* deletion in myeloid cells had reduced viability when exposed to high doses of LPS, to mimic conditions of sepsis (87). Like KLF4, KLF2 also outcompetes pro-inflammatory transcription factors such as NF-κB for the co-factor p300 (86–88). Together these observations indicate that KLF2 regulates programs that supress inflammatory activation and the absence of KLF2 in macrophages leads to greater antibacterial properties but also increases the unwanted side-effects of unrestrained inflammatory activation, as seen in sepsis.

Unlike KLF2 and KLF4, KLF6 is a pro-inflammatory transcription factor which is rapidly expressed after M1-type activation and suppressed in M2-type macrophages (73). In similar but opposing mechanisms to those described for KLF2 and KLF4, KLF6 activates the expression of pro-inflammatory genes through co-operation with NF-κB. In addition, KLF6 actively represses M2- polarisation in collaboration with PPARγ, although the exact mechanism by which this occurs is unclear (73). ChIP-PCR analysis revealed that KLF6 represses *Prdm1* which normally induces *Bcl6*. BCL6 is known for repressing pro-inflammatory cytokines and keeping monocytes quiescent, and thus the indirect repression of BCL6 by KLF6 results in the activation and polarisation of M1-type macrophages (89). In contrast, a study using myeloid *Klf6* deficient mice to study aortic dissection and intramural haematoma found that these mice had increased inflammatory macrophages in diseased aortic tissue and a lack of KLF6 resulted in increased levels of secreted GM-CSF (CSF2) (90).

Overall, there is a large body of data suggesting both activating and repressive activity of the macrophage expressed KLFs. And while some of these reports are conflicting, we posit that the majority of this data support the idea that KLF2 and KLF4 favour M2 polarisation and KLF6 favours M1 polarisation (see **Figure 5**).

### 4.3 What about the repressors?

In macrophages, repressive complexes are essential in preventing the expression of potent pro-inflammatory molecules when they are not required. As previously outlined, KLF2, KLF3, KLF4, and KLF6 have all been implicated in either promoting or inhibiting inflammatory responses. It is likely that these factors compete for occupancy of CACCC-box motifs in myeloid-specific promoters and enhancers, as has been reported in other KLF-regulated cell systems (20, 44, 91).

There is conflicting evidence as to how KLF4 influences macrophage activation (72, 84, 85). Some of these differing reports may in fact be due to the downstream actions of KLF4 target genes such as *Klf3*, and negative and positive feedback networks. *Klf3*-knockout mice are more sensitive to LPS treatment, and in the absence of this repressor, pro-inflammatory genes are more highly expressed (74, 92). Perhaps some of the effects attributed to KLF4 are mediated through repression by KLF3. KLF3 can repress pro-inflammatory gene expression via directly repressing transcription of the NF-κB p65 subunit (RelA) (74). KLF3 is also known to repress the *Lgals3* gene which stops the expression of Galectin-3, a metabolic protein known to regulate TGF-β signalling which in turn polarises macrophages to an anti-inflammatory M2-type (93). The role of the repressive KLFs (KLF3, KLF8 and KLF12) on the regulation of pro-inflammatory gene expression is worth further exploration.

### 4.4 Trained immunity

While not as clearly defined as that within the adaptive immune system, innate immune cells are also capable of developing immune memory (94). Rather than a clonal expansion of specifically reactive cells, trained immunity is driven by epigenetic modifications (95). These modifications keep certain genes accessible to TFs so they can be rapidly expressed following subsequent infection. Likewise, other genes are silenced to limit the adverse effects of chronic inflammation, such as tissue damage (96, 97). Trained immunity has been described in macrophages (98); however, it is not clear what TFs are responsible for these epigenetic alterations, nor is it known what factors are required to remove these modifications and return the cells to their pre-inflammatory state. Moreover, little is known about how KLF TFs may play a role in these mechanisms.

Trained immunity in mouse alveolar macrophages (AMs) *in vivo* has been linked to increased *Klf4* expression, and a high association of KLF4 binding within open chromatin regions (99). This also correlated with increased overall numbers of AMs, and with a more M2-like phenotype. Other KLF genes have been reported as differentially expressed in LPS-tolerised mouse BMDMs (98). KLF10, for example, is reported as an upregulated gene in tolerised BMDMs that have received a secondary treatment of LPS (98). However, Zhang et al. examined *Klf10*-deficient BMDMs and did not find them to have altered LPS-mediated endotoxin tolerance (100). These limited reports provide evidence that KLF4 could be involved in the epigenetic changes associated with gene priming or silencing during trained immunity, however there are vast knowledge gaps in this field and more work is needed to explore the role of other macrophage-expressed KLFs in this process.

## 5 KLFs in inflammatory disease

### 5.1 Regulation of pro-inflammatory cytokines

Most pro-inflammatory cytokines are short-lived and act locally at the site of infection or injury. They can alter the microenvironment, and signal to recruit inflammatory cells of both the innate and adaptive immune systems (2). IL-12 and IL-1β contribute to the pathogenesis of several inflammatory diseases, including inflammatory bowel disease, arthritis, psoriasis, lupus, and others (101–103). High expression of IL-12 (which is formed by a heterodimer of IL-12p35 and IL-12p40 subunits) is detected in skin lesions from patients with psoriasis and chronic atopic dermatitis (104). Treatment with blocking antibodies targeting the IL-12p40 subunit can be effective in treating these conditions (105). At the gene level, CACCC-motifs within the human *IL12A* (encoding the p35 subunit) promoter are essential for the expression of this gene following LPS-treatment (106). Additionally, *in vivo* footprinting of the *IL12B* (encoding the p40 subunit) promoter identified the GA-12 cis regulatory module that contains a CACCC-motif that is responsible for its repression (107). This region is protected by tightly bound chromatin in resting cells but it ‘opens’ following LPS/IFNγ stimulation (107). Given what we know about the KLF repressors and their ability to recruit epigenetic modifiers, KLF3, KLF8 and/or KLF12 may be involved in the chromatin remodelling that safeguards these sites from activation during homeostasis and LPS tolerance.

IL-1β has a well-established role in autoinflammation, and high levels of IL-1β can lead to symptoms resembling septic shock and multi-organ failure (108). IL-1β is kept in the cytosol as inactive pro-IL-1β, which allows for its early release following an inflammatory signal (109). Cleavage into its functional form requires caspases which are activated as part of inflammasome signalling complexes (110). Defective inflammasome signalling leads to conditions such as Familial Mediterranean Fever and Cryopyrin-associated Periodic Syndromes (CAPS) (111–113). High IL-1β is also linked to systemic and skin inflammation (114). Indeed, patients with inflammasome conditions, due to gain-of-function mutations in the cytosolic inflammasome-triggering PRRs NLRP3 or PYRIN, have very high levels of circulating IL-1β, which is associated with fever and skin rashes. Mutations in the *NLRP3* promoter region have been identified in a patient with CAPS. The promoter has a repressive CACCC-element 9bp down from the mutated site (115). While these authors speculated that there was a yet to be identified CACCC-binding repressor TF, whose binding and repressive influence was disrupted by this mutation, they did not investigate the KLF factors specifically. Thus, further work is needed to uncover the roles of KLF repressors in these and other contexts, and the important function of the KLF repressors in the pathogenesis of various acute and chronic inflammatory conditions should be investigated in more detail.

### 5.2 Gut inflammation

**Figure 6:**
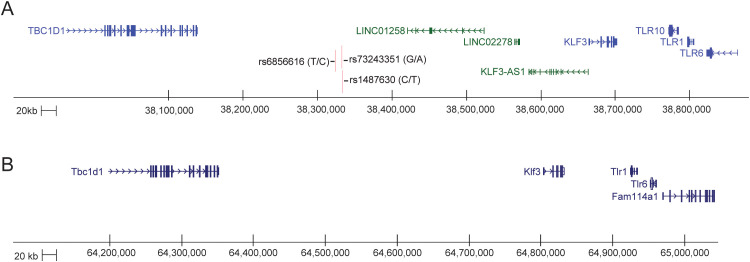
The KLF3 -TLR loci in human and mouse. **(A)** Schematic generated from the UCSC Genome Browser (GRCh38/hg38). The *KLF3* gene sits just upstream and is expressed in the opposite direction to three TLR genes, *TLR10*, *TLR1* and *TLR6*, and sits downstream of the *TBC1D1* gene within a 1Mb region of DNA on chromosome 4p14. There are a large number of lncRNAs (green) and enhancer signatures in this region between the coding genes, including KLF3-AS (121). There are three SNPs in the vicinity of one of these spliced LNC RNAs that are associated with inflammatory bowel disease (119, 120). The entire region is syntenic with mouse chr5qC3.1. **(B)** Schematic generated from the UCSC Genome Browser (CRCm38/mm10). The *Klf3* gene sits just upstream and is expressed in the opposite direction to two TLR genes, *Tlr1* and *Tlr6*, and sits downstream of the *Tbc1d1* gene within a 1Mb region of DNA on chromosome 5qC3.1.

To investigate the role of KLF4 and other KLFs in macrophage-driven inflammation, much focus has centred on their interactions with the NF-κB TF. Such studies have revealed a positive feedback loop between KLF4 and NF-κB which can be dysregulated in oesophageal and intestinal inflammation (116, 117) (**Figure 1E**). Indeed, analysis of GWAS studies linked to inflammatory bowel disease (IBD) has uncovered an association with the dysregulation of genetic feedback loops in macrophages and susceptibility to IBD (76, 118). Furthermore, several susceptibility loci for Crohn's disease: rs6856616, rs73243351 are located at 4p14, near *KLF3* and three TLR genes (119, 120) (**Figure 6**). Interestingly, *Klf3* is highly expressed in gut macrophages compared with other tissue macrophages (**Figure 5**). Ghaleb et al. describe a pro-inflammatory role for KLF4 in the intestinal cells of a dextran sodium sulphate (DSS) induced colitis mouse model (117). Mice with an intestinal-cell specific deletion of *Klf4* were significantly less sensitive to DSS-induced colitis and showed greater cell proliferation. Treatment with DSS activated the NF-κB signalling pathway in the colons of WT mice but not *Klf4*-deleted mice. This study highlights a pro-inflammatory role for KLF4 in a model of ulcerative colitis and shows that the pro- or anti-inflammatory roles for KLF4 are cell-type and tissue specific. As *KLF3* is a well-established target of KLF4, this raises the possibility that certain SNPs associated with Crohn's, or other IBDs, may exist in CREs near *KLF3* that disrupt its activation.

### 5.3 Psoriasis

Chronic inflammatory skin disorders, such as atopic dermatitis (AD) and psoriasis, are caused by a combination of impaired skin barrier formation and dysregulated immune cell function (122–125). KLF4 is critical for skin barrier formation (39) and aberrant expression has been linked to psoriasis (126, 127). *Klf4* ^-/-^ mice fail to form an intact skin barrier and die soon after birth (39). Skin barrier formation can be accelerated by treatment with corticosteroids, which induce gene expression changes that highly correlate with KLF4 overexpression in mouse models (128). Indeed, overexpression of KLF4 in combination with corticosteroid treatment resulted in even further accelerated skin barrier formation in mice (128). Several key transcriptional targets of KLF4 have been identified through over-expression and gene knockout studies (128). One of the genes identified was *Klf3.* This was shown to be upregulated when KLF4 was overexpressed and downregulated when KLF4 was deleted. Moreover, transactivation assays demonstrated that KLF4 was able to activate the *Klf3* promoter region, further confirming that *Klf3* is a direct KLF4 target gene (128) and also highlighting a cell intrinsic action of KLFs in keratinocytes.

*Klf2* ^+/-^ mice are more sensitive to chemical induced skin inflammation (129). On the other hand, *Klf6* deletion in macrophages results in reduced TPA-induced cutaneous inflammation and reduced cytokine gene expression (89). These studies demonstrate a network of activating and repressing KLFs that cooperate to fine-tune inflammatory gene expression. In addition, there is a strong possibility of interactions between dermal macrophages and keratinocytes that are driven by KLFs. Once again, conditional gene knockouts in different cell types of mice will help resolve skin cell intrinsic versus immune system functions for KLFs in psoriasis and other inflammatory skin disorders.

## 6 Future directions

Although we know a lot about how KLF4 and family members regulate gene expression to drive macrophage differentiation, M1-M2 polarisation, and activation of inflammatory genes, there is still much learn about mechanisms. We need more studies of expression changes of KLFs and their target genes at frequent time points in response to different stimuli such as those undertaken in some of the FANTOM experimental systems (76). Low coverage RNA-seq at a large number of time points to micro-dissect dynamic changes in inflammatory responses in carefully perturbed systems would advance our understanding of KLF networks. We need to try to perturb well studied systems (*e.g.* LPS-TLR4 responses in BMDM) at different time points in a dynamic way to try to tease apart requirements for initiation of the inflammatory response from maintenance and ultimate dampening of the response. Systems biology approaches to analysis of these datasets will be valuable. Some of the confusion in the literature about whether KLF4 acts as a repressor or activator of inflammatory gene expression likely comes from limitations inherent in the current genetic systems to study gain and loss of function, and in the design of specific experiments. In the future it might be informative to use degron tags of endogenous KLFs to rapidly deplete them at different stages of inflammatory responses to determine whether they play different roles and collaborate with different partners at different stages of immune responses.

It will also be very useful to determine what expression changes are direct or indirect consequences of loss of a particular KLF. Genetic deletion of KLF4 could well result in loss of expression of *Klf3* and other KLFs, which likely results in secondary changes in downstream shared target genes (*i.e.* disruption of an incoherent feed-forward loop, IFFL). So, ChIP-seq for KLFs at different stages of inflammatory responses in macrophages will be informative, as it has been for erythroid cells (20). ChIP-seq for other transcription factors such as IRFs, NF-κB and PU.1 in the presence and absence of KLF4 or other KLFs would help determine their inter-dependence for activation of key target genes; *i.e.* if or how they work together (**Figure 1E**).

Not all inflammatory signals are the same. There has been a large focus on LPS-TLR4 responses, but alternative models that activate different TLRs should be examined. There may be important differences in signalling and downstream activation of KLFs and their targets by engagement with different PAMPs. There has been a limited amount of work on post-translational modifications of KLFs in response to cytokine signalling and TLR signalling in macrophages. KLF4 and family members are phosphorylated, acetylated, ubiquitinated and SUMOylated in macrophages as they are in other systems, and these modifications are likely to influence function in important ways via degradation, shuttling between the nucleus and cytoplasm, and recruitment of different co-factors.

Transcription factors have been considered very hard to target therapeutically. This is certainly true for the KLF family. However, it is possible to target enzymes that induce PTMs in KLFs. MEK/ERK inhibitors, CtBP inhibitors, and PRMT5 inhibitors all have the potential to change PTMs in KLFs and thereby modify their function. Unfortunately, these enzymes act in many different signalling pathways and on many different TF targets, so inhibitors tend to be very nonspecific. Similarly, inhibitors of epigenetic writing and erasing activities of KLF-recruited epigenetic modifiers is theoretically possible. Bromo domain, P300/CBP, and HDAC inhibitors are all likely to effect KLF-dependent epigenetic effector mechanisms, but all are likely to be very non-specific. Finding ways to specifically target KLF functions with small molecule inhibitors or alternative methods (e.g. stable anti-sense RNA approaches) remains a challenge for the field.
